# Occurrence of parasites in waters used for crops irrigation and vegetables from the Savannah of Bogotá, Colombia

**DOI:** 10.1007/s11356-024-33088-1

**Published:** 2024-04-27

**Authors:** Daniel Eduardo Ospina Santos, Yulieth Alexandra Upegui Zapata, Catherine Aguilar Buitrago, Geraldine Sánchez Herrera, Libia Eunise Chandillo Becoche, Myriam Consuelo López Páez, Martha Cristina Bustos López, Carolina Ortiz Pineda

**Affiliations:** 1 Maestría en Ciencias Microbiología, Ciudad Universitaria, Universidad Nacional de Colombia, Bogotá, DC CP 111321 Colombia; 2 Departamento de Salud Pública, Facultad de Medicina, Ciudad Universitaria, Universidad Nacional de Colombia, Bogotá, DC CP 111321 Colombia; 3 Programa de Bacteriología y Laboratorio clínico, Universidad Colegio Mayor de Cundinamarca, Bogotá, DC CP 111051 Colombia; 4 Departamento de Ingeniería Civil y Agrícola, Facultad de Ingeniería, Ciudad Universitaria, Universidad Nacional de Colombia, Bogotá, DC CP 111321 Colombia

**Keywords:** Agricultural irrigation, Vegetables, Food safety, Parasites, Colombia, Health risk

## Abstract

The World Health Organization (WHO) has established as a criterion of parasitological quality for irrigation water, ≤ 1 helminth egg/liter, which guarantees the safety in agricultural products. In this study, the presence of parasites in surface water used for irrigation of crops (*n* = 96) and vegetables (celery and lettuce) (*n* = 120), from the Former La Ramada irrigation district, was evaluated using conventional and molecular parasitological methods. Our findings showed contamination of irrigation systems in the study area with domestic wastewater, demonstrated by the presence of Ancylostomatidae eggs, *Ascaris* spp., *Hymenolepis* spp., *Trichuris* spp., *Capillaria* spp., *Giardia* spp. cysts, and oocysts of *Toxoplasma gondii* and *Cryptosporidium* spp. A prevalence of 33% and 23.3% was calculated for helminths and protozoa, respectively in vegetables, representing a possible risk to human and animal health in relation to these parasites. These findings show the need for continuous monitoring of the water quality used for crop irrigation, as well as the safety of food, taking into account the values established in national and international regulations.

## Introduction

Water is an essential input for agricultural production and plays a fundamental role in food security (Mabhaudhi et al. 2016). According to The Food and Agriculture Organization (FAO 2017), agriculture uses 70% of freshwater withdrawals worldwide, with the use of urban effluents for crop irrigation being a very common practice (Almuktar et al. 2018; Pedrero et al. 2010). The quality of irrigation water differs by region, country and location, depending on local sanitation conditions. In some countries, crop irrigation is carried out with treated wastewater, while in others, where sanitation conditions and treatment systems are poor or non-existent, contaminated water is used without any control (Jiménez et al. 2016).

In Colombia, less than 40% of wastewater is treated, then raw wastewater or wastewater diluted with surface water is used for irrigation, generating possible risks to public health, especially when used for the production of food for direct consumption (Ofori et al. 2021). The regulations and guidelines around the world regarding agricultural water reuse are principally human-health focused, unsatisfactory concerning some of the possible hazardous contaminants such as emerging pollutants (including the parasites), and with large discrepancies when compared with each other (Shoushtarian and Negahban-Azar 2020).

Several studies have reported the risk faced by consumers of agricultural products that have been irrigated with wastewater or contaminated sources (Dickin et al. 2016, Amoah et al. 2018a, Khan et al. 2022), and it has even been estimated that about 70% of diarrhea cases are caused by the consumption of food contaminated with chemical and/or biological agents, including parasites (Kirk et al. 2017; Gizaw 2019). Foodborne parasites constitute one of the most representative and relevant etiological groups in public health, mainly in developing countries (Food and Agriculture Organization of the United Nations 2014; Rodriguez-Morales et al. 2016). This is generated not only by the use of effluents contaminated with wastewater for irrigation crops, but also by the inappropriate handling of fruits and vegetables during their production chain (cultivation, distribution, processing, packaging and sale) (Briñez et al. 2012; Olea et al. 2012).

This study evaluates the occurrence of parasites in waters used for crop irrigation and vegetables from the Savannah of Bogotá, Colombia. We assess the parasitological water quality coming from the middle basin of the Bogotá River, in the area of the irrigation district of La Ramada, where due to the economic growth of the region, raw domestic and industrial wastewater is also discharged directly into the irrigation channels (Argüello and Bustos 2018). Vegetables such as lettuce and celery were analyzed, using parasitological and PCR assay methods, for contamination with helminth and protozoan parasites endemic in Colombia and important in the Savannah of Bogotá, Colombia.

## Materials and methods

### Study area and sample collection

The study was carried out in the Former La Ramada irrigation and drainage district, located in the municipalities of Mosquera and Funza (Cundinamarca), Colombia. This district is responsible for providing permanent irrigation and drainage services for water from the middle basin of the Bogotá River through hydraulic infrastructure works (Corporación Autónoma Regional de Cundinamarca (CAR) 2010; Corporación Autónoma Regional de Cundinamarca (CAR) 2011), which extend through the municipalities of Mosquera, Tenjo, Funza, Cota, Madrid and Bojacá.

**Fig. 1:**
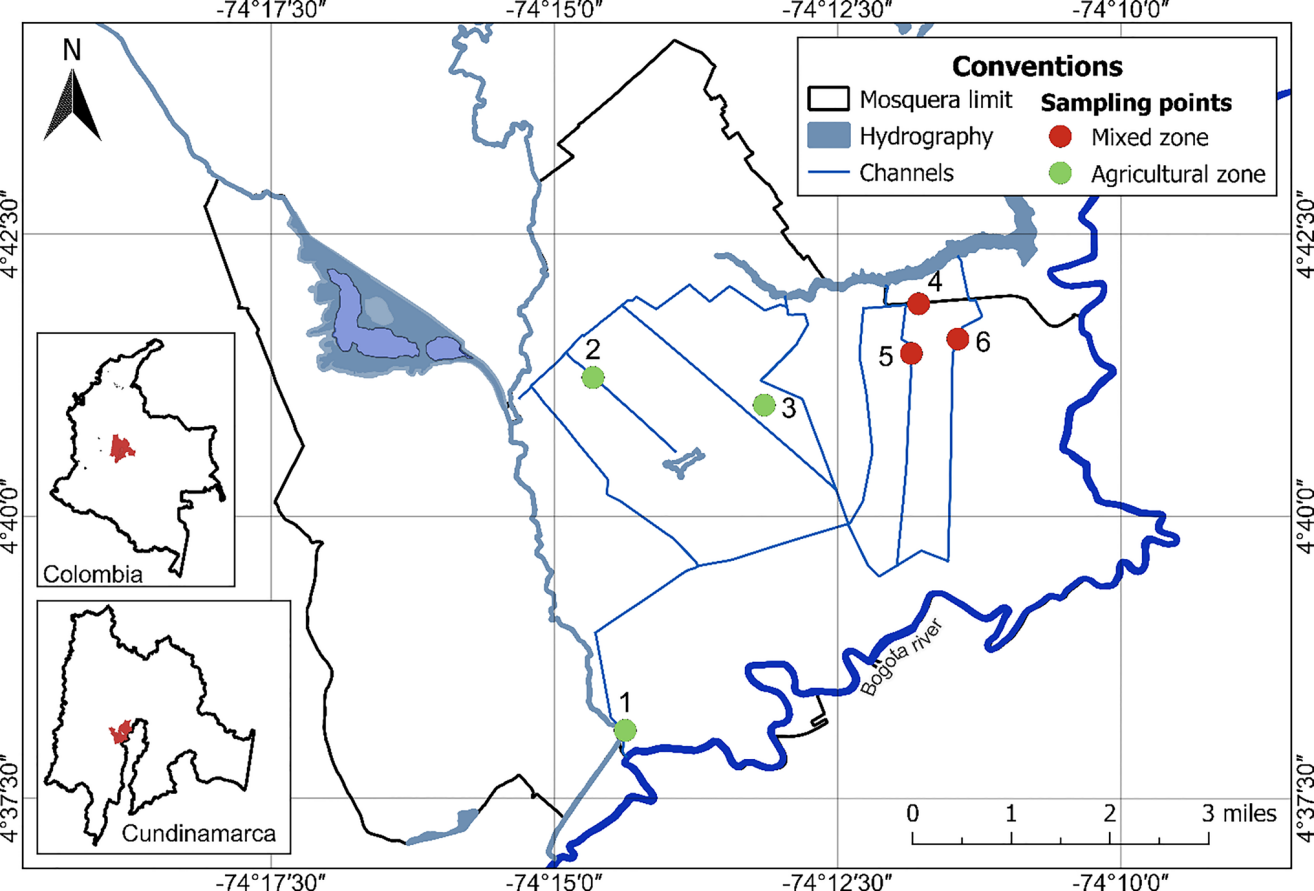
Geographical distribution of the sampled sites of water used for irrigation and vegetables (celery and lettuce), from the Former La Ramada irrigation district

Water sampling was taken in six sampling sites from irrigation canals of the Former La Ramada district (Fig. 1): site 1: longitude: − 74.240309, Latitude: 4.634444; site 2: longitude: − 74.196457, Latitude: 4.698042; site 3: longitude: − 74.218966, Latitude: 4.683125; site 4: longitude: − 74.197369, longitude: 4.690720, site 5: longitude: − 74.196457, Latitude: 4.698042 and site 6: longitude: − 74.190544, Latitude: 4.692900. Sites one, two, and three were selected because of their predominant agricultural activity, along with animal husbandry, primarily cattle, pigs, and poultry (Fig. 1 Agriculture Zone). Sites four to six had a high influence of industrial, agricultural and residential activities. At these sites, a total of 96 water spot samples were collected during the months of June to November 2019; 60 samples in the dry season during the months of July, August, and September to 2019, when irrigation water is used all the time for cultivation, and 36 samples in the rainy season during May, June, October, and November of 2019, when irrigation water was used less.

Each sample, comprising a minimum of 2 L of water was collected, transported in a clean plastic container, washed previously with Tween 80 to avoid the adhesion of parasitic structures and stored at 4 °C. The maximum processing time was 96 hours after collection.

A total of 120 samples of fresh vegetables (lettuce (*n* = 108) and celery (*n* = 12)) were collected, during harvest at Site 1, Site 3 and Site 4, because they were irrigated with the irrigation canal waters. Those vegetables are eaten raw, they have a high consumption by the local population, large volume of trade and consumption in Bogota city and availability at all times of the year. A completely randomized design was used, selecting 3 farms in each chosen site, Three plants were taken in each farm for a total of 9 plants per site, and the sampling unit was made up by quartering. A total of eight samplings were conducted during the years 2019 and 2021 in both the rainy and dry season.

### Detection and enumeration of parasitic forms in irrigation water samples

For the detection and enumeration of helminth eggs in the water, the samples were processed using the method of Bailenger (1979) modified by Ayres and Mara (1997). Once the concentration of each sample was completed, it was observed using brightfield microscopy with a McMaster Chamber.

### Molecular detection of protozoa in irrigation water

The direct centrifugation methodology described in the Environmental Protection Agency (EPA) method 1693 (EPA 2014) was used. The sediment obtained was subjected to DNA extraction using the Fast DNA Spin Kit for soil Cat: 116560200 MP Biomedicals, with the aid of the Super FastPrep-2MT instrument (MP Biomedicals)*,* following the manufacturer’s instructions*.* The recovered DNA was quantified using NanoDrop™ One- ThermoScientific and stored at − 20 °C until analysis.

Molecular detection (Table 1) was performed individually for each protozoan species using qPCR with different sets of specific primers and probes reported by Mejía et al. (2013) for *Giardia* spp., using the Biorad kit; *C. cayetanensis* as reported by Ortiz et al. (2020), using the Applied biosystems 7500. For the identification of *T. gondii* oocysts, the primers described by Opsteegh et al. (2010) were used, slightly modified for amplification detection using the Sybr select master mix thermo kit, for which 10 μL of the mix, 1 μL of each primer (final concentration 0.5 μM) and 3 μL of the sample were used, for a final volume of 15 μL. DNA extracted from cat feces donated by Dr. João Luis Garcia (Universidad de Londrina (UEL) Paraná, Brazil) was used as a positive control for this parasite. For *Cryptosporidium* spp. the methodology described by Burnet et al. (2013) was used. DNA from *C. cayetanensis* and *Cryptosporidium* spp. donated by Dr. Ana Luz Galván from the Universidad de Antioquia, (Medellín-Colombia) were used as controls. In the case of *Giardia* spp. DNA was obtained from culture in TYS-33 medium (Keister 1983).

**Table 1:** qPCR setup for the detection of *Giardia* spp.*, T. gondii, Cryptosporidium* spp. and *C. cayetanensis*

SETUP	*Giardia* spp.	*T. gondii*	*Cryptosporidium* spp.	*C. cayetanensis*
Forward (5′-3′)	CATGCATGCCCGCTCA	AGGAGAGATATCAGGACTGTAG	GTTTTCATTAATCAAGAACGAAAGTTAGG	ATGTTTTAGCATGTGGTGTGGC
Reverse (3′-5′)	AGCGGTGTCCGGCTAGC	GTCGTCTC GTCTAGATCG	GAGTAAGGAACAACCTCCAATCTCTAG	GCAGCAACAACAACTCCTCATC
Probe (5′-3′)	6FAM/AGGACAACGGTTGCAC/MGB	Syber Green	Syber Green	HEX-TACATACCCGTCCCAACCCTCGA-BHQ1
Primer (μM)	1.6	0.5	0.5	0.5
Probe (μM)	0.1	Syber green Mix	Syber green Mix	0.15
Amplicon Size (bp)	88	162	107	141
CYCLES				
*G. duodenalis*				
Parameter	Initial denaturalization		40 Amplification Cycles	
			Denature	Anneal/extend
Temp.	50 (°C)	95 (°C)	95 (°C)	60 (°C)
t (mm:ss)	2:00	10:10	0:15	1:00
*T. gondii; Cryptosporidium* spp. y *C. cayetanensis*				
Parameter	Initial denaturalization		45 Amplification Cycles	
			Denature	Anneal/extend
Temp.	95 (°C)		95 (°C)	60 (°C)
t (mm:ss)	3:00		0:15	0:30

### Physicochemical and microbiological characteristics of irrigation water samples

Physicochemical (pH, dissolved oxygen (DO), conductivity and temperature) and microbiological (most probable number (MPN) of total coliforms and *Escherichia coli*) parameters were measured in water samples used for crop irrigation. In each sample, a multiparameter DO / pH / Conductivity (HACH HQ40D) was used. Following the manufacturer’s instructions, the measurement of the physicochemical parameters was carried out in situ.

For the identification of microbiological parameters, the methodology described in the Standard Methods for the Examination of Water and Wastewater (9221) was followed (APHA et al. 2017). The procedure was performed by calculating the MPN of coliforms and *E. coli.* Both physicochemical and microbiological results were analyzed with GraphPad prism v5.0.

### Detection of parasites in vegetable samples

Thirty grams (30 g) of each type of directly consumed vegetable (celery or lettuce) were taken, and the methodology described by Matosinhos and collaborators (Matosinhos et al. 2016) was followed. Due to the large amount of organic detritus, an additional step was performed, to allow the visualization of protozoan cysts and oocysts using the Formol-Ether Concentration Method. Following the technique described by Triviño-Valencia et al. (2016), this sample was observed using light microscopy to characterize helminth eggs and larvae, along with protozoa (*Entamoeba* spp., *Endolimax* spp., and others). Furthermore, detection of *Giardia* spp. cysts and *Cryptosporidium* spp. oocysts was performed by immunofluorescence, using the A100FLK Aqua-Glo™ G/C Direct Comprehensive Kit (Waterborne Inc.®) following the manufacturer’s instructions. The criteria for positivity are based on the parameters defined in EPA method 1623.1 (EPA 2014; Palacios 2017).

## Results

### Detection of helminth eggs and larvae in irrigation water by microscopy

It was found that 68% (65/96) of the samples were positive for helminth eggs. Ancylostomatidae was found in 42/96 (43.75%) of the samples and *Ascaris* spp. was found in 33/96 (34.37%) of the samples were the predominant parasites with averages of 1.53 and 0.81 eggs per liter (eggs/L) respectively. Followed by *Hymenolepis* spp. was found in 11/96 of the samples (11.45%), *Trichuris* spp. were observed in 5/96 of the samples (5.20%) and *Capillaria* spp. was found in 2/96 of the samples (2.08%) with averages of 0.22, 0.02 and 0.008 eggs/L respectively. Nematode larvae were observed (52.08%) with an average of 1.9 nematode larvae per liter of water.

The highest parasite load was found at site 6. The predominant parasites were *Ascaris* spp. With 93.75% (2.6 eggs/L), *Hymenolepis* spp. 62.5% (1.23 eggs/L), *Trichuris* spp. With 31.25% (0.17 eggs/L) and nematode larvae with 81.25% (4.05 larvae/L). In the waters analyzed at Site 1, the presence of Ancylostomatidae predominated with 68.75% (5.54 eggs/L). Site 4 showed a high presence of nematode larvae with 68.8% (1.9 larvae/L).

### Molecular detection of protozoa in irrigation water

Molecular detection of *Giardia* spp. was 10.41% (10/96), *T. gondii* 18.75% (18/96) and *Cryptosporidium* spp. 52% (32/56). *C. cayetanensis* was not detected by qPCR (Table 2). According to geographical location, all the sites showed contamination with *T. gondii* and *Cryptosporidium* spp. The waters from the Urban-Industrial (mixed) sites 5 and 6 showed the highest contamination with *Giardia* spp. at 7.29% (7/96), compared to agricultural sites 1 and 3 with 3.12% (3/96). For *Cryptosporidium* spp., a very similar prevalence was demonstrated between the study areas, ranging from 6.5% to 9.7%. For *T. gondii,* agricultural sites 1 and 2 showed the highest prevalence of this parasite with 4.2% each.

**Table 2:** Molecular detection by qPCR of *Giardia* spp., *T. gondii, Cryptosporidium* spp. and *C. cayetanensis* in irrigation water from the Former La Ramada district

Sampling Points	Positive number (Percentage) [Wet/Dry season positive samples]					
	Land	Number examined [Wet/Dry Season]	*Giardia* spp.	*T. gondii*	*Cryptosporidium* spp.	*C. cayetanensis*
1	A	16 [6/10]	2 (2.0) [2/0]	4 (4.1) [3/1]	5 (8.1) [3/2]	0 (0) [0/0]
2	A	16 [6/10]	0 (0) [0/0]	4 (4.1) [3/1]	5 (8.1) [4/1]	0 (0) [0/0]
3	A	16 [6/10]	1 (1.0) [1/0]	3 (3.1) [2/1]	6 (9.7) [4/2]	0 (0) [0/0]
4	I	16 [6/10]	0 (0) [0/0]	2 (2.0) [1/1]	4 (6.5) [3/1]	0 (0) [0/0]
5	I	16 [6/10]	1 (1.0) [0/1]	3 (3.1) [2/1]	6 (9.7) [4/2]	0 (0) [0/0]
6	I	16 [6/10]	6 (6.2) [4/2]	2 (2.0) [1/1]	6 (9.7) [4/2]	0 (0) [0/0]
TOTAL		**96** [36/60]	**10 (10.4)** [7/3]	**18 (18.7)** [12/6]	32 (51.6) * [22/10]	**0 (0)** ^**^ [0/0]

Data represent the percentage of intestinal parasites in irrigation water collected on both A (Agricultural) and I (urban-industrial) land. * *n* = 62 **n = 88

Due to resource limitations and sample volume, a specific number of samples were selectively evaluated for *Cryptosporidium* spp. and *C. cayetanensis*. Of the 96 samples available, 62 water samples were evaluated for *Cryptosporidium* and 88 samples were evaluated for *C. cayetanensis*.

Considering the season of sample collection, the presence of parasites was higher in the rainy season with 68.3%, with a higher prevalence of *Cryptosporidium* spp. followed by *T. gondii* and *Giardia* spp. (Table 2).

### Identification of physicochemical and microbiological parameters in irrigation water samples

Table 3 shows the average values of physicochemical and microbiological data for each of the 6 sampling points. The data show higher MPN values for total coliforms and *E. coli* in the area with purely agricultural activity. Likewise, higher average pH and temperature values are observed in this same zone. In contrast, it was found that the zone with mixed activity (agricultural, urban-industrial), obtained a higher average value of dissolved oxygen (DO) and conductivity.

**Table 3:** Average values of physicochemical and microbiological parameters of irrigation water used in the Former La Ramada irrigation district

Sampling Sites	MPN TOTAL COLIFORMS/100 mL	MPN *E. coli*/100 mL	DO (mg/LO_2_)	EC (μS/cm)	pH	Temperature (°C)
1	5.57E × 10^4^	3.03 × 10^4^	0.92	804.31	6.87	13.63
2	4.04 × 10^4^	1.04 × 10^4^	0.55	643.00	6.69	13.43
3	5.24 E × 10^3^	1.87 × 10^3^	0.82	718.69	6.83	14.09
4	4.35 × 10^6^	4.08 × 10^5^	1.61	946.19	7.00	15.28
5	2.52 × 10^6^	1.55 × 10^6^	5.15	687.13	7.11	15.71
6	6.41 × 10^7^	2.88 × 10^7^	1.47	951.50	7.37	17.12

*MPN* most probable number, *DO* dissolved oxygen, *EC* electrical conductivity

### Detection of parasites (protozoa and helminths) in vegetables

**Fig. 2:**
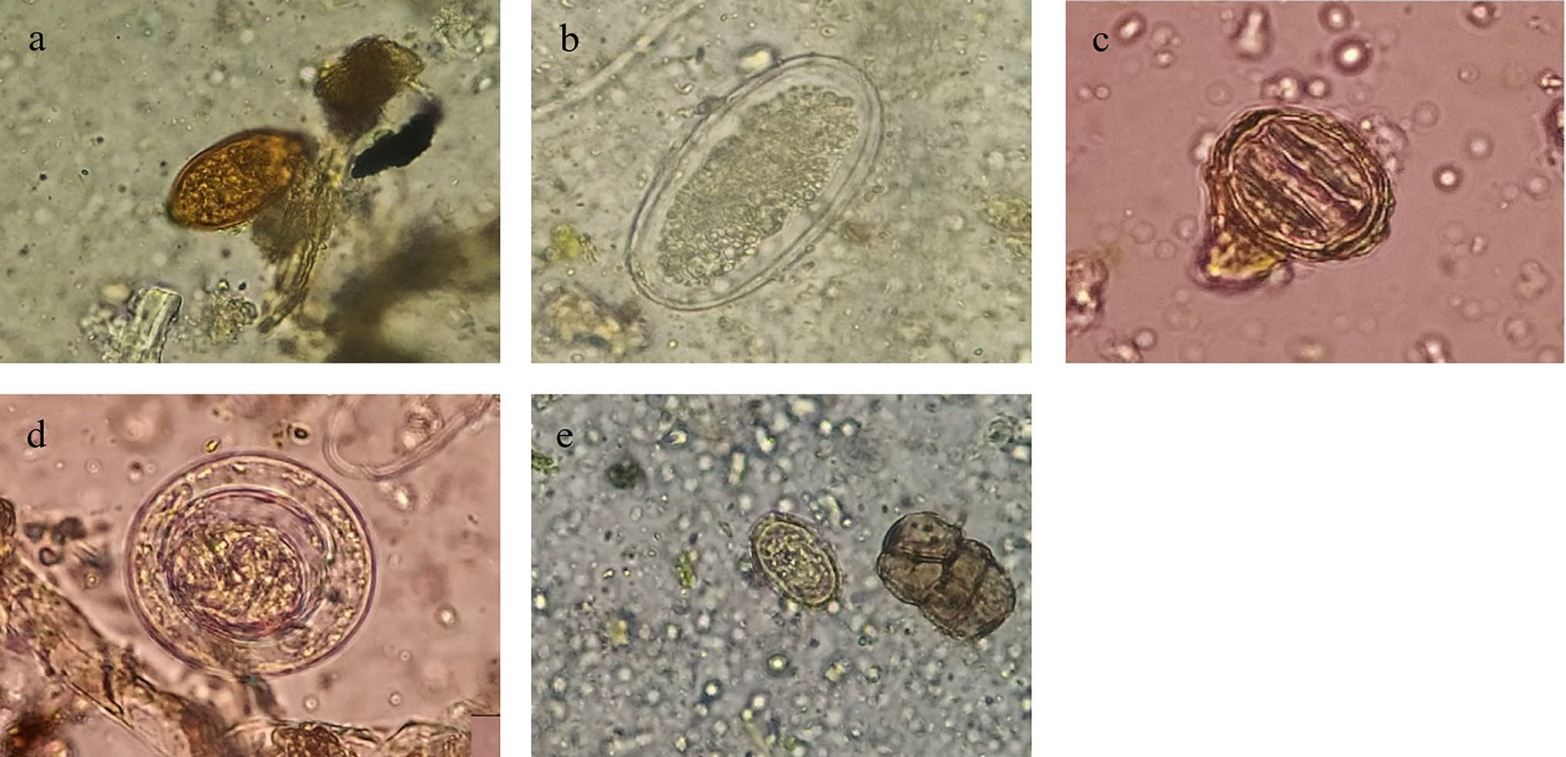
Helminth eggs detected in samples of vegetables irrigated with water from the farmer La Ramada district. *Trichuris* spp. (**a**- site1); Ancylostomatidae (**b**-site3); *Ascaris* spp. (**c**-site1); *Hymenolepis* spp. (**d**-site1); and *Capillaria* spp. (**e**-site1)

The detection of parasites in vegetables grown in the Former La Ramada area showed that 49.16% (59/120) of the samples (lettuce and celery) were positive for at least one helminth egg, cyst or protozoan oocyst (Table 4). The prevalence of helminths and protozoa detected in the 120 vegetable samples showed that 33.33% (40/120) of the vegetables were positive for helminth eggs, with *Ascaris* spp. being the most prevalent parasite, with an average of 0.43 eggs/30 g of sample analyzed, followed by Ancylostomatidae, *Hymenolepis* spp., *Trichuris* spp. and *Capillaria* spp. (Fig. 2). Of the samples, 23.33% (28/120) presented some protozoa, with *Entamoeba* spp. being the most representative, detected with an average of 0.45 cysts/30 g, followed by *Giardia* spp., *Cryptosporidium* spp. and *Endolimax* spp. (Table 4).

**Table 4:** Prevalence of intestinal parasites in vegetable samples (Lettuce and Celery)

Parasites		Former La Ramada district (*n* = 120)			
		Pos.	Prev.	X̄	Min-Max
Eggs	*Ascaris* spp.	33	27.50%	0.433	0–8
	Ancylostomatidae	11	9.16%	0.142	0–4
	*Hymenolepis* spp.	2	1.66%	0.017	0–1
	*Trichuris* spp.	2	1.66%	0.017	0–1
	*Capillaria* spp.	1	0.83%	0.008	0–1
Cysts	*Entamoeba* spp.	18	15%	0.458	0–17
	*Giardia* spp.	9	7.50%	0.125	0–3
	*Endolimax* spp.	1	0.83%	0.008	0–1
Oocysts	*Cryptosporidium* spp.	4	3.33%	0.058	0–3

Pos (Positive samples); Prev. (Prevalence); X̄ (average value of helminth eggs and cysts or oocysts of protozoa in 30 g of plant material analyzed); Min-Max (Minimum and maximum values of parasites detected in vegetables)

*Entamoeba* spp. were detected in the three points evaluated, with a sample of lettuce in which 17 cysts were reported (site 3). Site 1 (*n* = 93) had the highest diversity of parasites; predominantly *Ascaris* spp., *Entamoeba* spp. and *Giardia* spp. The data also showed that 7.5% (9/120) of the vegetables were contaminated with both helminths and protozoa; 5.83% (7/120) of these samples were collected at site 1; the sample with the highest diversity of parasites had 4 Ancylostomatidae eggs, 1 *Capillaria* spp. egg and 3 *Cryptosporidium* spp. oocysts (Table 5).

**Table 5:** Prevalence of parasites detected at three sampling sites in the former La Ramada irrigation and drainage district

Parasites		Site 1 (*n* = 93)				Site 3 (*n* = 21)				Site 4 (*n* = 6)			
		Pos	Prev.	X̄	Min-Max	Pos	Prev.	X̄	Min-Max	Pos	Prev.	X̄	Min-Max
Eggs	*Ascaris* spp.	33	35.4%	0.55	0–8	0	0%	–	–	0	0%	–	–
	Ancylostomatidae	7	7.57%	0.11	0–4	4	19.0%	0.28	0–3	0	0%	–	–
	*Hymenolepis* spp.	2	2.15%	0.02	0–1	0	0%	–	–	0	0%	–	–
	*Trichuris* spp.	2	2.15%	0.02	0–1	0	0%	–	–	0	0%	–	–
	*Capillaria* spp.	1	1.07%	0.01	0–1	0	0%	–	–	0	0%	–	–
Cysts	*Entamoeba* spp.	12	12.9%	0.27	0–6	4	19.0%	0.28	0–3	2	33.3%	3.83	0–17
	*Giardia* spp.	7	7.52%	0.13	0–3	2	9.52%	0.09	0–1	0	0%	–	–
	*Endolimax* spp.	1	1.07%	0.01	0–1	0	0%	–	–	0	0%	–	–
Oocysts	*Cryptosporidium* spp.	4	4.30%	0.07	0–3	0	0%	–	–	0	0%	–	–

Pos (Positive samples); Prev. (Prevalence); X̄ (average value of helminth eggs, cysts or oocysts of protozoa in 30 g of plant material analyzed); Min-Max (Minimum and maximum values of parasites detected in vegetables)

During the rainy season, both helminths (16.90% (12/71)) and protozoa (23.94% (17/71)) were observed. For the dry season, a prevalence of 57.14% (28/49) of helminths and 22.44% (11/49) of protozoa was recorded, thus reporting higher prevalence of these parasites for this season, except for the genus *Entamoeba* spp. which presented a higher prevalence in the rainy season (Table 6).

**Table 6:** Prevalence of parasites detected in vegetables collected in rainy and dry seasons

Parasite		Rainy (*n* = 71)				Dry (*n* = 49)			
		Pos	Prev.	X̄	Min-Max	Pos	Freq.	X̄	Min-Max
Eggs	*Ascaris* spp.	10	14.08%	0.25	0–8	23	46.93%	0.69	0–3
	Ancylostomatidae	2	2.81%	0.02	0–1	9	18.36%	0.30	0–4
	*Hymenolepis* spp.	1	1.40%	0.01	0–1	1	2.04%	0.02	0–1
	*Trichuris* spp.	1	1.40%	0.01	0–1	1	2.04%	0.02	0–1
	*Capillaria* spp.		0%	–	–	1	2.04%	0.02	0–1
Cysts	*Entamoeba* spp.	12	16.90%	0.52	0–17	6	12.24%	0.36	0–6
	*Giardia* spp.	5	7.04%	0.12	0–3	4	8.16%	0.12	0–2
	*Endolimax* spp.	0	0%	–	–	1	2.04%	0.02	0–1
Oocysts	*Cryptosporidium* spp.	1	1.40%	0.01	0–1	3	6.12%	0.12	0–3

Prev. (Prevalence); X̄ (average value of helminth eggs, cysts or oocysts of protozoa in 30 g of plant material analyzed); Min-Max (minimum and maximum values of parasites detected in vegetables)

**Fig. 3:**
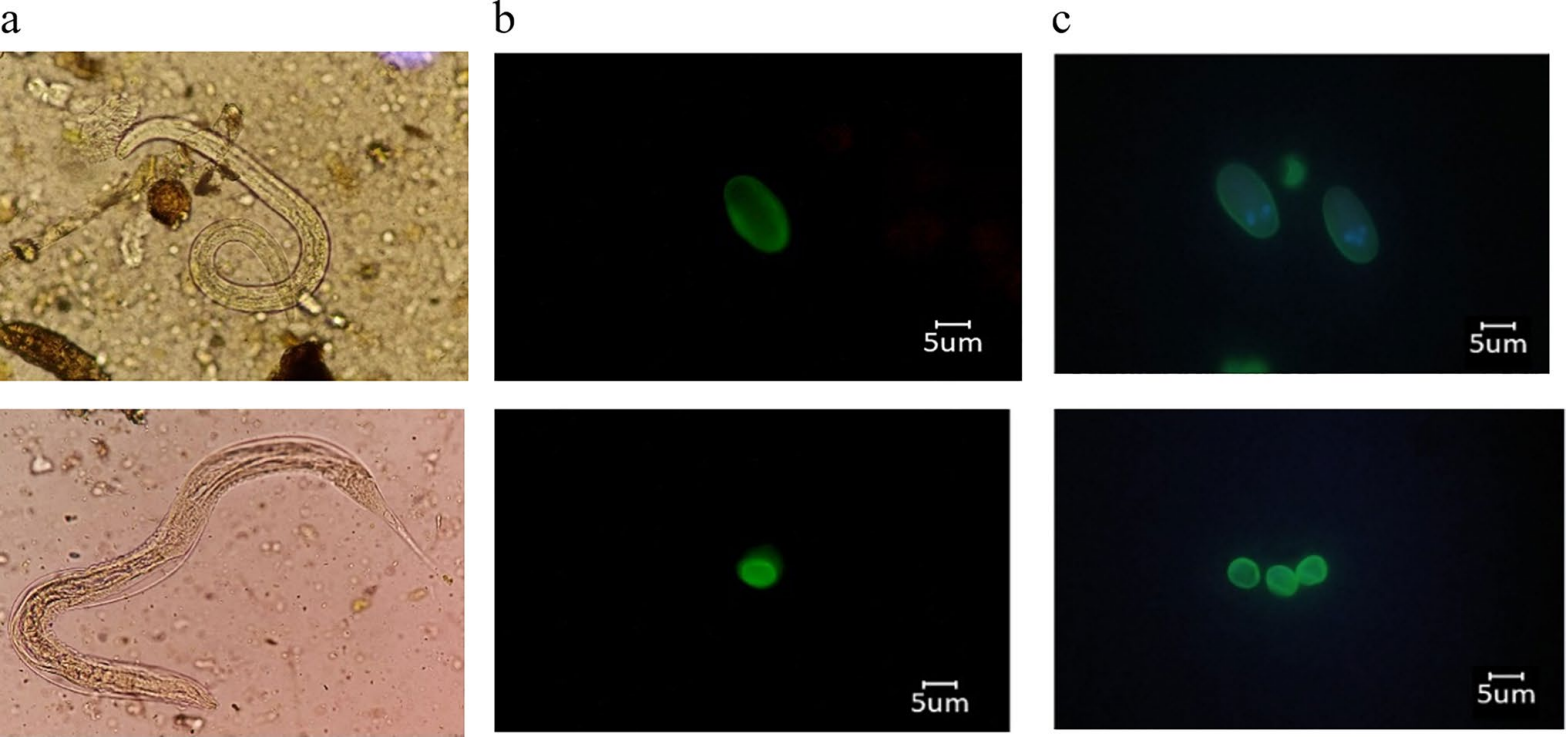
Detection of intestinal parasites in vegetables irrigated with water from the farmer La Ramada irrigation district. Panel **a**: Nematode larvae detected in vegetable samples, bright field microscopy observation. Panel **b**: *Giardia* spp. cysts (b) and *Cryptosporidium* spp. oocysts (b) by immunofluorescence (FITC filter). Panel **c**: Positive control of *Giardia* spp. cysts (c) and *Cryptosporidium* spp. oocysts (c) by immunofluorescence (DAPI filter)

Nematode larvae were observed in 39.16% (47/120) of the samples, with an average of 1.68 larvae/30 g of sample analyzed, with site 1 showing the highest values of these parasitic stages (Fig. 3A). Immunofluorescence assays detected the presence of 4 samples positive for *Cryptosporidium* spp. oocysts and 7 for *Giardia* spp. cysts (Fig. 3B).

## Discussion

Foodborne parasites have long been a “neglected” group of pathogens, attributed to vulnerable or low-resource populations (Robertson 2018). However, in both developed and developing countries, there have been numerous cases of outbreaks associated with the consumption of fresh produce, which have increased considerably (Kopper et al. 2009; Ryan and Cacciò 2013; Carstens et al. 2019). The variety and types of microorganisms found in these foods (especially vegetables) vary substantially, depending on several factors such as the type of crop, region of origin, growth conditions, handling and transport circumstances, among others.

In Colombia, the Ministry of Health reported in 2021, 603 foodborne disease (FBD) outbreaks, with an estimated 6883 affected people (Instituto Nacional de Salud 2022). Although those reports broadly classify the etiologic agents that can cause FBDs, it is evident from those reports that the *Entamoeba* genus is the gastrointestinal protozoa most commonly associated with outbreaks and food contamination, after viruses and bacteria (Varela et al. 2016; INS 2018; Montañez et al. 2020). The real impact of these infections and the true number of outbreaks caused by these parasites are unknown and underestimated in Colombia, due to the lack of mandatory reporting and the absence of an active surveillance system at the national level (INS 2018).

In this study, different parasitological and molecular techniques were used to evaluate the quality of water used for agriculture and some vegetables (lettuce and celery) crop in this zone. These methodologies allowed the detection of protozoa and helminths in food crops and water samples. The results showed that the water sources being used for crop irrigation in this area of the country do not comply with the guidelines established by the World Health Organization regarding their parasitological quality (should have less than 1 egg/L to guarantee human health safety). For example, in sampling site 6, the average for *Ascaris* spp. Was 2.6 eggs/L and for *Hymenolepis* spp. 1.23 eggs/L and for site 1 the average for Ancylostomatidae was 5.54 eggs/L. The presence of helminth and protozoan has also been found in the food (lettuce and celery) grown in this area. 33.33% (40/120) of the vegetables were positive for helminth eggs, with *Ascaris* spp. being the most prevalent parasite, with an average of 0.43 eggs/30 g of sample analyzed, followed by Ancylostomatidae, *Hymenolepis* spp., *Trichuris* spp. and *Capillaria* spp.

In the review of the literature from Colombia, there are no studies with these same characteristics that have simultaneously evaluated the parasitological quality of water used for agriculture and food irrigated with this water. According to the physicochemical and microbiological characteristics of the water used for irrigation analyzed in this study (Table 3), the results of positivity for *Giardia* cysts *(*10.41%), *Cryptosporidium* oocysts (51.62%) and *T. gondii* (18.75%) obtained by qPCR and the occurrence of different helminths are not surprising, since fecal contamination of these effluents is evident.

As can be seen in Table 3, the sites considered in the agricultural zone have anoxic conditions and high electrical conductivity values. In the mixed zone conductivity remains high despite higher DO values, which is a clear indicator of the discharge of wastewater of both industrial and domestic origin, affecting the development of both biological activity and degradation processes (Abella and Martínez 2014).

In line with the findings of this current study, in Morocco, Amahmid et al. (2022) found crops contaminated with various levels of parasite eggs and cysts due to irrigation with raw sewage. Lettuce samples were positive (27.7%) for one or more helminth eggs including the pathogens *Ascaris* and *Trichuris* with an average of 4.7 eggs/kg. These values differ from the current study where 33.33% (40/120) of the vegetables were positive for helminth eggs, with *Ascaris* spp. being the most prevalent parasite, and an average of 0.43 eggs/30 g, followed by Ancylostomatidae, *Hymenolepis* spp., *Trichuris* spp. and *Capillaria* spp. The dissimilarities observed in these studies may be a result of the level of environmental sanitation in different countries. Moreover, in our research, in zones where the hygienic settings of the irrigation water canal structure were not suitable, the occurrence of eggs, cysts and larvae parasites in vegetables was apparent.

In Mexico, Chaidez et al. (2005) evaluated the presence of *Giardia* spp. and *Cryptosporidium* spp. in 58 irrigation water samples and found that 48.2% and 50% of the samples were positive, respectively. Results coincide with our study where the characteristics of the agricultural areas were similar.

It is worth noting the importance of this study’s finding on *T. gondii* prevalence (18.75%), which is one of the most prevalent protozoa in the water samples studied and, according to published reports, is the main cause of outbreaks in Latin America (Rosado-García et al. 2017; Minuzzi et al. 2021).

The presence of *Cryptosporidium* spp. oocysts in 51.62% (32/62) of the water samples evaluated is not surprising, since these results are consistent with documented studies around the world, where the presence of this parasite is found in water used for crop irrigation, as documented in Greece and United Kingdom (Spanakos et al. 2015; Bodley-Tickell et al. 2002).

The research developed by Palacios (2017) determined the presence of *Cryptosporidium* spp. in calf feces and water samples (Ecuador). The author showed that in calves there is a prevalence of 93.3% of *Cryptosporidium* spp. (112 positive cases out of 120), while in water, a concentration of 5 oocysts/100 mL was determined. Based on these findings, the researcher concluded that the high prevalence of *Cryptosporidium* spp. in calves may be the cause of the presence of this parasite in water.

Colombia is a tropical country with dry and rainy seasons. The contamination of irrigation channels may be related to runoff in rainy seasons, and to the discharge of untreated domestic water during the dry season, among other causes (Steele and Odumeru 2004; Almuktar et al. 2018).

Referring to the evaluation of the presence of helminth eggs, it was found that Ancylostomatidae (43.75%) and *Ascaris* spp. (34.37%) were the most prevalent genera in irrigation water samples. These results are in agreement with the findings of Amoah et al. (2018b) in South Africa, Hajjami et al. (2012) in Morocco, Ortiz and López (2012) in Colombia and Trang do et al. (2006) in Vietnam.

Regarding the concentration of eggs, previous studies have reported total values of 0.1 to 3 helminth eggs per liter of untreated water, and 0.1 to 1 viable helminth egg per liter of treated water in the Savannah of Bogotá (Campos-Pinilla et al. 2008; Campos et al. 2018). Our results reported average numbers ranging from 0 to 5.54 eggs/L and an average of 1.9 nematode larvae per L. These results suggest that these samples are not in compliance with WHO quality criteria guidelines (Campos et al. 2018).

Of the vegetable samples investigated (lettuce and celery), 49.16% (59/120) were positive for at least one helminth and/or protozoan, with *Ascaris* spp. (27.5%) being the most prevalent helminth and *Entamoeba* spp. (15%) the most frequently observed protozoan. These results are varying to those obtained at different latitudes. For example, in Venezuela, *Ascaris* spp. (11.81%) and *Cryptosporidium* spp. (5.51%) were detected when 127 vegetable samples were examined (Cazorla et al. 2009). In Minas Gerais (Brazil), Luz et al. (2017) evaluated 108 vegetable samples, obtaining a contamination percentage of 50.9% (55/108), predominantly nematode larvae (36.5%), *Entamoeba coli* cysts (26.0%) and Ancylostomatidae eggs (12.9%). In Italy, Caradonna et al. (2017), evaluated ready-to-eat salads from industrial and local brands, finding a prevalence of 0.6% for *G. duodenalis*, 0.8% for *T. gondii*, 0.9% for *Cryptosporidium* spp. and 1.3% for *C. cayetanensis*. In Norway, Robertson et al. (2006) demonstrated the presence of *Giardia* and *Cryptosporidium* in fruits and vegetables. In Ethiopia, Alemu et al. (2020) evaluated different vegetables, finding that, 87 (25.1%) of these were contaminated with at least one parasite species.

## Conclusions

In conclusion, this study establishes that there is a potential risk of transmission of parasites of public health interest from the consumption of vegetables grown in the Savannah of Bogotá, Colombia. Molecular detection showed that the most relevant parasite was *Cryptosporidium* spp. and in terms of geographic region, the industrial and urban region had the highest concentration of *Giardia* spp. The detection of DNA of *T. gondii*, *Giardia* spp. and *Cryptosporidium* spp. in addition to the observation of different helminth eggs in water samples used for irrigation and vegetables, shows that fecal contamination of effluents (with animal and human feces) is evident. Therefore, it is necessary to inform stakeholders -farmers, consumers, regulatory entities- to carry out a risk analysis of the production chain of food grown in this area of the country, based on Good Agricultural Practices (GAPs), to implement appropriate preventive measures to reduce the risk of contamination and possible transmission of these parasites.

It is recommended to carry out viability tests to obtain more robust data on the risk that these parasites represent for the community.

To our knowledge, this is the first investigation describing *T. gondii* detection in irrigation water samples collected from the territory of Savannah of Bogotá, Colombia using sensitive molecular tools.

## Data Availability

The authors declare that the data supporting the findings of this study are available within the paper. Should any raw data files be needed in another format they are available from the corresponding author upon reasonable request. Source data are provided with this paper.

## References

[CR1] Abella G, Martínez C (2014). Contribution of a tributary stream to eutrophication of Lake Tota (Boyacá, Colombia). Rev Colomb Quim.

[CR2] Alemu G, Nega M, Alemu M (2020). Parasitic contamination of fruits and vegetables collected from local markets of Bahir Dar city, Northwest Ethiopia. Res Rep Trop Med.

[CR3] Almuktar S, Abed S, Scholz M (2018). Wetlands for wastewater treatment and subsequent recycling of treated effluent: a review. Environ Sci Pollut Res.

[CR4] Amahmid O, Asmama S, Bouhoum K (2022). Pathogenic parasites in sewage irrigated crops and soil: pattern of occurrence and health implications. Int J Environ Health Res.

[CR5] Amoah ID, Adegoke AA, Stenström TA (2018). Soil-transmitted helminth infections associated with wastewater and sludge reuse: a review of current evidence. Trop Med Int Health.

[CR6] Amoah ID, Reddy P, Seidu R (2018). Removal of helminth eggs by centralized and decentralized wastewater treatment plants in South Africa and Lesotho: health implications for direct and indirect exposure to the effluents. Environ Sci Pollut Res.

[CR7] Baird R, Bridgewater L (2017) Standard methods for the examination of water and wastewater. 23rd edition. American Public Health Association, Washington, DC

[CR8] Argüello H, Bustos M (2018). Contamination by Pathogenic Microorganisms in Water used for Agricultural Irrigation on the Sabana de Bogotá, Colombia. Adv J Food Sci Technol.

[CR9] Ayres R, Mara D (1997). Análisis de aguas residuales para su uso en agricultura. Manual de Técnicas parasitológicas y bacteriológicas de laboratorio. (OMS, Issue 1).

[CR10] Bailenger J (1979). Mechanisms of parasitological concentration in coprology and their parasitical consequences. Am J Med.

[CR11] Bodley-Tickell AT, Kitchen SE, Sturdee AP (2002). Occurrence of Cryptosporidium in agricultural surface waters during an annual farming cycle in lowland UK. Water Res.

[CR12] Briñez K (2012). Calidad del agua para consumo humano en el departamento del Tolima. Facultad Nacional de Salud Pública.

[CR13] Burnet JB, Ogorzaly L, Tissier A, Penny C, Cauchie HM (2013). Novel quantitative TaqMan real-time PCR assays for detection of *Cryptosporidium* at the genus level and genotyping of major human and cattle-infecting species. J Appl Microbiol.

[CR14] Campos MC, Beltrán M, Fuentes N, Moreno G (2018). Helminth eggs as parasitic indicators of fecal contamination in agricultural irrigation water, biosolids, soils and pastures. Biomédica.

[CR15] Campos-Pinilla C, Cárdenas-Guzmán M, Guerrero-Cañizares A (2008). Comportamiento de los indicadores de contaminación fecal en diferente tipo de agua de la Sabana de Bogotá (Colombia). Univ Sci.

[CR16] Corporación Autónoma Regional (2010) Carta ambiental. Edición No 25 - ISSN-0213. 7. https://sie.car.gov.co/server/api/core/bitstreams/6a4641fa-9559-4959-bba9-96c611e3e4b9/content. Accessed 5 Sept 2023

[CR17] Corporación Autónoma Regional (2011) Producto final – Anexo No. 24 La Ramada. https://www.car.gov.co/uploads/files/5aeb755bef54b.pdf. Accessed 10 Sept 2023

[CR18] Caradonna T, Marangi M, Del Chierico F, Ferrari N, Reddel S, Bracaglia G, Normanno G, Putignani L, Giangaspero A (2017). Detection and prevalence of protozoan parasites in ready-to-eat packaged salads on sale in Italy. Food Microbiol.

[CR19] Carstens C, Salazar JK, Darkoh C (2019). Multistate outbreaks of foodborne illness in the United States associated with fresh produce from 2010 to 2017. Front Microbiol.

[CR20] Cazorla D, Morales P, Chirinos M, Acosta ME (2009). Evaluación parasitológica de hortalizas comercializadas en Coro, estado Falcón, Venezuela. Bol Mal Salud Amb.

[CR21] Chaidez C, Soto M, Gortares P, Mena K (2005). Occurrence of *Cryptosporidium* and *Giardia* in irrigation water and its impact on the fresh produce industry. I Int J Environ Health Res.

[CR22] Dickin SK, Schuster-Wallace CJ, Qadir M, Pizzacalla K (2016). A review of health risks and pathways for exposure to wastewater use in agriculture. Environ Health Perspect.

[CR23] EPA (2014) Method 1693: Cryptosporidium and giardia in disinfected wastewater by concentration/IMS/IFA. Federal Register. EPA 821-R-R14-013. U.S. Environmental Protection Agency, Washington, DC

[CR24] Food and Agriculture Organization (2017) Water for sustainable food and agriculture. A report produced for the G20 Presidency of Germany. https://www.fao.org/3/i7959e/i7959e.pdf. Accessed 12 Sept 2023

[CR25] FAO/WHO (Food and Agriculture Organization of the United Nations and World Health Organization) FAO Headquarters; Rome Italy (2014) Multicriteria-Based Ranking for Risk Management of Food-borne Parasites. http://www.fao.org/3/a-i3649e.pdf. Accessed 15 Sept 2023

[CR26] Gizaw Z (2019). Public health risks related to food safety issues in the food market: a systematic literature review. Environ Health Prev Med.

[CR27] Hajjami K, Ennaji MM, Fouad S, Oubrim N, Cohen N (2012) Wastewater reuse for irrigation in Morocco: helminth eggs contamination′s level of irrigated crops and sanitary risk (a case study of Settat and Soualem regions). J Bacteriol Parasitol 04. 10.4172/2155-9597.1000163

[CR28] INS (2018). Enfermedades Transmitidas por Alimentos Colombia 2017.

[CR29] Instituto Nacional de Salud (2022) Boletin Epidemiologico Semanal, Semana epidemiológica 04 del 23 al 29 de enero de 2022. 1–28. https://www.ins.gov.co/buscador-eventos/BoletinEpidemiologico/2022_Boletin_epidemiologico_semana_4.pdf. Accessed 18 Sept 2023

[CR30] Jiménez B, Maya C, Velásquez G, Torner F, Arambula F, Barrios JA, Velasco M (2016). Identification and quantification of pathogenic helminth eggs using a digital image system. Exp Parasitol.

[CR31] Keister DB (1983). Axenic culture of *Giardia lamblia* in TYI-S-33 medium supplemented with bile. Trans R Soc Trop Med Hyg.

[CR32] Khan MM, Siddiqi SA, Farooque AA, Iqbal Q, Shahid SA, Akram MT, Rahman S, Al-Busaidi W, Khan I (2022). Towards sustainable application of wastewater in agriculture: a review on reusability and risk assessment. Agronomy.

[CR33] Kirk MD, Angulo FJ, Havelaar AH, Black R (2017). Diarrhoeal disease in children due to contaminated food. Bull World Health Organ.

[CR34] Kopper G, Calderón G, Schneider S, Domínguez W, Gutiérrez G (2009) Enfermedades transmitidas por alimentos y su impacto socioeconómico. Estudios de caso en Costa Rica, El Salvador, Guatemala, Honduras y Nicaragua. Informe técnico sobre ingeniería agrícola y alimentaria Organización de las Naciones Unidas para la Agricultura y la Alimentación 1:1–194. https://www.fao.org/3/i0480s/i0480s00.pdf. Accessed 18 Sept 2023

[CR35] Luz JGG, Barbosa MV, Carvalho AG, Resende SD, Dias JVL, Martins HR (2017). Contamination by intestinal parasites in vegetables marketed in an area of Jequitinhonha Valley, Minas Gerais, Brazil. Rev Nutr.

[CR36] Mabhaudhi T, Chibarabada T, Modi A (2016) Water-food-nutrition-health nexus: linking water to improving food, nutrition and health in sub-Saharan Africa. Int J Environ Res Public Health 13. 10.3390/ijerph13010107 10.3390/ijerph13010107 PMC4730498 26751464

[CR37] Matosinhos FC, Valenzuela VC, Silveira JA, Rabelo EM (2016). Standardization of a method for the detection of helminth eggs and larvae in lettuce. Parasitol Res.

[CR38] Mejía R, Vicuña Y, Broncano N, Sandoval C, Vaca M, Chico M, Cooper PJ, Nutman TB (2013). A novel, multi-parallel, real-time polymerase chain reaction approach for eight gastrointestinal parasites provides improved diagnostic capabilities to resource-limited at-risk populations. Infect Genet Evol.

[CR39] Minuzzi CE, Fernandes FD, Portella LP, Bräunig P, Sturza DAF, Giacomini L, Salvagni E, Ribeiro JDS, Silva CR, Difante CM, Farinha LB, Menegolla IA, Gehrke G, Dilkin P, Sangioni LA, Mallmann CA, Vogel FSF (2021). Contaminated water confirmed as source of infection by bioassay in an outbreak of toxoplasmosis in South Brazil. Transbound Emerg Dis.

[CR40] Montañez T, Novoa M, Sánchez L, Ortiz C (2020). Parásitos protozoarios transmitidos por alimentos ¿Cómo estamos en colombia?. Biociencias..

[CR41] Ofori S, Puškáčová A, Růžičková I, Wanner J (2021). Treated wastewater reuse for irrigation: pros and cons. Sci Total Environ.

[CR42] Olea A, Díaz J, Fuentes R, Vaquero A, García M (2012). Foodborne disease outbreaks surveillance in Chile. Rev Chilena Infectol.

[CR43] Opsteegh M, Langelaar M, Sprong H, den Hartog L, De Craeye S, Bokken G, Ajzenberg D, Kijlstra A, van der Giessen J (2010). Direct detection and genotyping of *toxoplasma gondii* in meat samples using magnetic capture and PCR. Int J Food Microbiol.

[CR44] Ortiz C, López MC y Rivas FA (2012) Prevalencia de helmintos en la planta de aguas residuales del municipio El Rosal, Cundinamarca. Revista de Salud Pública 14, 296–304. 10.1590/s0124-00642012000200010 10.1590/s0124-00642012000200010 23250372

[CR45] Ortiz C, Temesgen TT, Robertson LJ (2020). Multiplex quantitative PCR analysis of strawberries from Bogotá, Colombia, for contamination with three parasites. J Food Prot.

[CR46] Palacios TE (2017). Prevalencia de *Cryptosporidium* spp. y *Giardia* spp. en terneros, y su presencia en agua y en niños con problemas digestivos en el cantón San Fernando, Ecuador. Maskana.

[CR47] Pedrero F, Kalavrouziotis I, Alarcon JJ, Koukoulakis P Asano T (2010) Use of treated municipal wastewater in irrigated agriculture-review of some practices in Spain and Greece. Agric Water Manag 97: 1233–1241. 10.1016/j.agwat.2010.03.003

[CR48] Robertson LJ (2018). Parasites in food: from a neglected position to an emerging issue. Adv Food Nutr Res.

[CR49] Robertson LJ, Hermansen L, Gjerde BK (2006). Occurrence of *Cryptosporidium* oocysts and *Giardia* cysts in sewage in Norway. Appl Environ Microbiol.

[CR50] Rodriguez-Morales AJ, Bolivar-Mejía A, Alarcón-Olave C, Calvo-Betancourt LS (2016). Parasites in food: illness and treatment. Encyclopedia of Food and Health.

[CR51] Rosado-García FM, Guerrero-Flórez M, Karanis G, Hinojosa MDC, Karanis P (2017). Water-borne protozoa parasites: the Latin American perspective. Int J Hyg Environ Health.

[CR52] Ryan U, Cacciò S (2013). Zoonotic potential of *Giardia*. Int J Parasitol.

[CR53] Shoushtarian F, Negahban-Azar M (2020). World wide regulations and guidelines for agriculturalwater reuse: a critical review. Water (Switzerland).

[CR54] Spanakos G, Biba A, Mavridou A, Karanis P (2015). Occurrence of *Cryptosporidium* and *Giardia* in recycled waters used for irrigation and first description of *Cryptosporidium parvum* and *C. Muris* in Greece. Parasitol Res.

[CR55] Steele M, Odumeru J (2004). Irrigation water as source of foodborne pathogens on fruit and vegetables. J Food Prot.

[CR56] Trang do T, van der Hoek W, Cam PD, Vinh KT, Hoa NV, Dalsgaard A (2006). Low risk for helminth infection in wastewater-fed rice cultivation in Vietnam. J Water Health.

[CR57] Triviño-Valencia J, Lora F, Zuluaga JD, Gomez-Marin JE (2016). Detection by PCR of pathogenic protozoa in raw and drinkable water samples in Colombia. Parasitol Res.

[CR58] Varela ZS, Perez L, Estrada D (2016). Bacteria causing of foodborne diseases: an overview at Colombia. Salud Uninorte.

